# Detection of gene expression changes at chromosomal rearrangement breakpoints in evolution

**DOI:** 10.1186/1471-2105-13-S3-S6

**Published:** 2012-03-21

**Authors:** Adriana Muñoz, David Sankoff

**Affiliations:** 1 School of Information Technology & Engineering, University of Ottawa, Ottawa, K1N 6N5, Canada; 2 Department of Mathematics & Statistics, University of Ottawa, Ottawa, K1N 6N5, Canada

## Abstract

**Background:**

We study the relation between genome rearrangements, breakpoints and gene expression. Genome rearrangement research has been concerned with the creation of breakpoints and their position in the chromosome, but the functional consequences of individual breakpoints remain virtually unknown, and there are no direct genome-wide studies of breakpoints from this point of view. A question arises of what the biological consequences of breakpoint creation are, rather than just their structural aspects. The question is whether proximity to the site of a breakpoint event changes the activity of a gene.

**Results:**

We investigate this by comparing the distribution of distances to the nearest breakpoint of genes that are differentially expressed with the distribution of the same distances for the entire gene complement. We study this in data on whole blood tissue in human versus macaque, and in cerebral cortex tissue in human versus chimpanzee. We find in both data sets that the distribution of distances to the nearest breakpoint of "changed expression genes" differs little from this distance calculated for the rest of the gene complement. In focusing on the changed expression genes closest to the breakpoints, however, we discover that several of these have previously been implicated in the literature as being connected to the evolutionary divergence of humans from other primates.

**Conclusions:**

We conjecture that chromosomal rearrangements occasionally interrupt the regulatory configurations of genes close to the breakpoint, leading to changes in expression.

## Background

The phenotypic consequences of genome rearrangements in humans, such as infertility or developmental pathologies when these mutations occur in the germ line, and cancer when they occur in somatic cells, are well documented [1] and often understood down to the level of changes in gene expression. The classic example is the Philadelphia t(9;22)(q34;q11) translocation creating the Philadelphia chromosome [2] and the BCR-Abl fusion gene whose tyrosine kinase product has wide-ranging molecular interactions ultimately responsible for chronic myeloid leukemia. The situation with the homozygotic rearranged genomes of reproductively isolated populations is quite different. The breakpoints of the evolutionary rearrangements differentiating these genomes are known to co-occur with a large number of genomic features, such as regions that are gene-rich regions, GC-rich, hypomethylated, duplicated, peri-centromeric or subtelomeric, as often reviewed (e.g., [3]), but the functional consequences of individual breakpoints remain virtually unknown, and there are few direct genome-wide studies of breakpoints from this point of view. An early comparison of the chimpanzee and human genomes [4] found genes on rearranged chromosomes tended to change expression, and included a report that some, unspecified, genes within 2 Mb of breakpoints, or in the same chromosomal band region, changed more than others, but this work was limited by the small set of breakpoints only known at that time from cytogenetic studies in the early 1980s [5].

In this paper, we propose a new paradigm for this type of investigation. The idea is basically to compare any changes of expression of genes that are close to, or even disrupted by, chromosomal breakpoints in the comparison of two genomes with changes affecting the gene complement more generally, controlled of course for tissue and experimental conditions. This is not a trivial exercise. There are now high-resolution techniques to identify breakpoint regions [6-8], and thousands of data sets containing the results of whole-genome microarray assays, but comparative, whole genome data sets, controlled for tissue, with orthologous chromosomal positions specified for two species, are not easy to come by [9].

We have been able to make use of two, relatively early, tissue-controlled comparisons of orthologs in humans and non-human primates, the first [10] on whole blood tissue in macaques and humans, and the second [11] on the cerebral cortex of chimpanzees and humans. The blood comparison lacks chromosomal positioning of genes, and does not examine chromosomal rearrangements. The cerebral cortex study relies on breakpoint data from early cytological studies only. Both suffer, for our purposes, from obsolete gene nomenclature. Although we have implemented a system for high throughput analysis, the largely manual conversion of gene names remains a bottleneck that will only be relaxed when more comparative expression data becomes available using current gene and marker terms.

In the next section, we first formalize the null hypothesis of no systematic relationship between gene expression and proximity to breakpoints. We then describe the ortholog expression data sets, the breakpoint data sets, and our protocol for linking the two, as well as the details of our method and its implementation. In the following section, we present the statistical results of our study on change of expression near breakpoints. We find little evidence for rejecting the null hypothesis in either the human-macaque whole blood tissue data set or the human-chimpanzee cerebral cortex dataset. For the few genes closest to breakpoints that do change expression, however, several have previously been tied to have some interesting correlates. Then, in the Conclusions, we discuss the potential for larger scale studies within this paradigm.

## Results

### The null hypothesis

Were there no association between breakpoint creation and change of expression of neighbouring genes, we would expect changed-expression genes to be spatially distributed independently of breakpoint positions. Consider the interval determined by the position *a*_1 _and *a*_2 _of the two breakpoints on either side of a changed-expression gene. Let *u *= |*a*_1 _- *a*_2_|/2. The position of the gene, considered as a random variable *y *should be uniformly distributed in the interval [*y_min_*, *y_min _*+ 2*u*] where *y_min _*= min(*a*_1_, *a*_2_). The distance *x *to the closest breakpoint will then be distributed as a uniform variable on the interval [0, *u*].

**Figure 1:**
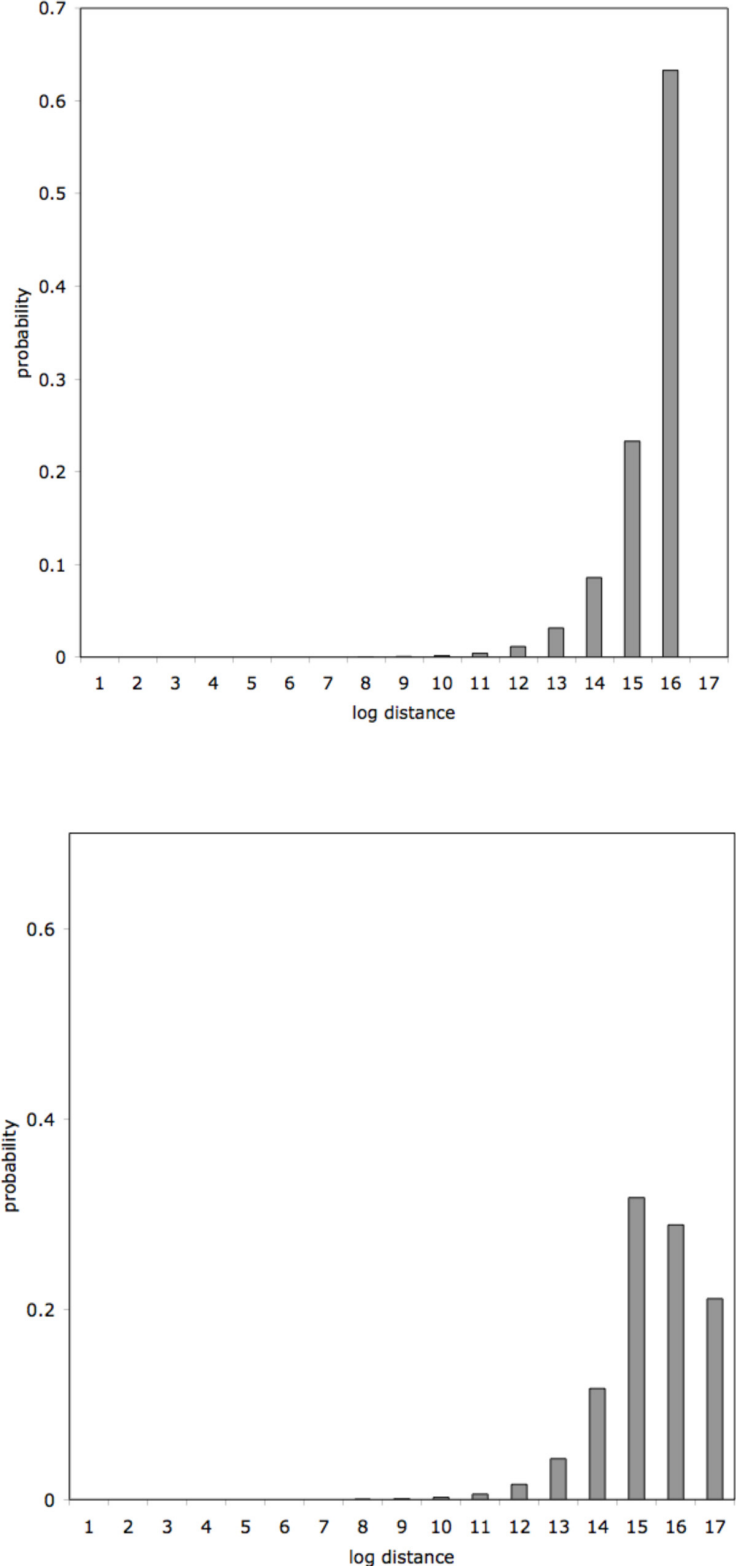
**Null hypotheses.** a) Distribution of *z *= log distance to nearest breakpoint, under the null hypothesis, with *u *= *e*^16^. b) Predicted empirical frequency distribution, based on equally weighted *u *= 15, 16, 17.

For visualization purposes, since the scale of intergenic distances is of the order of hundredths or thousandths of inter-breakpoint distances, we will study the distribution of *z *= log *x *rather than of *x*. Since *x *is uniform on [1, *u*], the probability density of *z *will have the form of a truncated positive exponential distribution

(1) $$p\left(z\right)=e^{z-u},$$

for 0 ≤ *z *≤ *u*, as in Figure 1a.

Since the distance 2*u *between the breakpoints will itself be distributed randomly (as the distance between two order statistics, namely a negative exponential) and depend on the length of the chromosome and the number of breakpoints, the empirical distribution of distances is predicted by a sum of variables, all with density *p*(*z*) but with different parameters *u*, as in Figure 1b.

### The data

To assess this hypothesis, we need to choose two genomes that are closely enough related that a comparison of gene expression still carries a signal of events in their recent evolutionary divergence, but distant enough so that there are numerous rearrangement breakpoints in their genomic alignment. For our purposes, we should also have relations of orthology established across the two genomes. Of the many expression databases available, there are few that satisfy these criteria. In the future, however, we can expect many more evolution-oriented genome-wide expression projects and this motivates our preliminary study. The present study is confined to two comparisons, one of the human and macaque genomes and gene expression in whole blood samples, and the second of human and chimp genomes and gene expression in cerebral cortex tissue. The tools we use to analyze these data, however, are applicable to much wider datasets.

#### The gene expression data

##### The gene expression data: whole blood tissue

Dillman et al. [10] analyzed whole blood tissue in human and three closely related non-human primates (NHP) namely the rhesus macaque, the cynomologous macaque, and the green african monkey. Each of their probe sets was defined by 54,000 probes, representing 38,500 genes from the completely sequenced human genome (2004 release).

The gene expression profiles for non-human primates (NHP) and human whole blood tissue were compared using a variety of statistical techniques (principal components, hierarchical clustering, analysis of variance) in order to find genes differentially expressed in humans and NHPs. The results include genetic elements identified as genes, mRNAs and ESTs.

Note that where these data tell us a gene is expressed more in one genome than the other, it does not tell us whether expression increased in the first genome since divergence from a common ancestor, or whether it decreased in the other.

We extracted 317 genetic elements with significant fold change from this table to use in testing our hypotheses. It is important to note that there is no gene coordinate or BPR information in the gene expression database. Thus, the crux of our investigation is to relate these unpositioned expression data associated with gene names to the breakpoint data, which is simply positional, with no gene names. To do so, we require a database containing both name and positions of human genes.

##### The gene expression data: cerebral cortex tissue

The second case study is confined to the comparison of the chimpanzee and human genomes and gene expression in brain tissue. It is based on a separate study by Cáceres et al. [11] where they analyzed brain tissue in human, chimpanzee and rhesus macaque using rhesus macaque as an outgroup.

Cáceres et al. [11] analyzed brain tissue in human, chimpanzee and rhesus macaque using rhesus macaque as an outgroup. They measured gene expression levels by using Affymetrix human microarrays. Each of their probe sets was defined by 12,625 probes, representing 10,000 genes.

We loaded the set of 80 differentially expressed genes that were up-regulated or down-regulated in chimp into the gene expression database as described in the preceding Section.

#### The breakpoint data

Breakpoints can today be determined more precisely than with the classical cytogenetics methods [6]. Comparing different human genomes, the position of the breakpoint can by determined down to the nucleotide level [12], but this is not generally for inter-specific comparisons where positional homology may not be well-defined especially for non-coding regions [13]. Lemaitre et al. [7] compared the genomes of human and five mammals: dog, mouse, rat, macaque and chimp, using a methodology that allowed them to delineate evolutionary breakpoint regions along the human genome with a finer resolution than observed previously.

These authors defined a breakpoint region (*BPR*) in the human genome as "a region that underwent at least one large chromosomal structural change, or is orthologous to such region in a non-human lineage".

They performed pairwise comparisons between human and the other mammals and identified 622 non-intersecting BPRs ranging from 1 to 2,887,673 nucleotides with a mean size of 104 kb. Those 622 BPRs are stored in a database of sets of coordinates of breakpoints, organized by chromosome, in the format of Table 1.

**Table 1:** Database of BPRs

Chromosome	Begin	End	Evolutionary branch
chr1	10382322	10382387	dog
chr1	109923784	109923788	chimp
chr1	143495190	143766399	macaque
chr1	144691208	144707142	primates
chr1	144850157	145574145	chimp
chr1	150079680	150138541	dog
chr3	126855424	127207816	primates
chr3	128287101	128299344	macaque
chr5	102756311	102787215	mouse
chr5	110090786	110287080	rodents
chr5	112304457	112304458	rat
chr22	34277622	34286037	rodents
chr22	37056914	37068605	rat

##### Breakpoints for the whole blood tissue study

**Figure 2:**
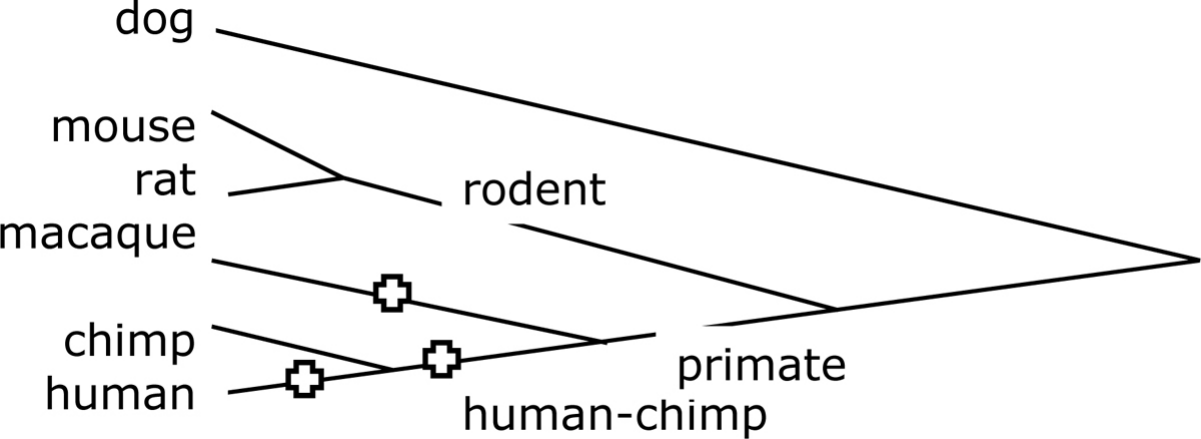
**Phylogenetic relationships of species.** Phylogeny of species in the breakpoint database, with branches pertinent to the human-macaque comparison indicated.

To compare the macaque genome to the human, we extracted only those breakpoints, 92 of them, on evolutionary branches leading to these species from their most recent common ancestor, namely those labelled in the dataset as human, human-chimp or macaque, as illustrated in Figure 2. All other breakpoints are found in both human and macaque or in neither.

It is important to note that there is no systematic accounting of gene expression or even of gene information in the BPR database, although these features of the human genome (but not in other genomes) played a role in the characterization of BPR regions in [7].

##### Breakpoints for the cerebral cortex study

There are too few breakpoints on the human genome during the period of evolutionary divergence from chimpanzee to be able to carry out our study, and the breakpoints in [7] are only given in terms of the human genome.

Thus, we identified chimpanzee-human orthologs using Biomart [14], and we ran the Cassis software [8] to identify 38 breakpoints on the chimp genome during the period of evolutionary divergence from humans. We then loaded this set of breakpoints into the breakpoint database as described in the preceding Section.

#### The genome database

Since the breakpoint data are stored in UCSC Genome Browser [15] coordinates, we used the entire set of human genes from the human genome assembly of May, 2004 (NCBI35 or hg17) from this browser as a baseline against which to test our differentially expressed genes in human and macaque whole blood tissue. This set is more comprehensive and more accurately positioned than the original set of "no changed expression" genes in the original two studies.

For our cerebral cortex study, we used the entire set of chimpanzee genes from the chimpanzee genome assembly of March, 2006 (CHIMP2.1) and the human genome assembly of February, 2009 (NCBI37 or GRCh37) from the Ensembl Genome Browser [16] as a basis for comparison of our differentially expressed genes.

#### Making connections

We first sketch the general protocol for linking each breakpoint dataset with the corresponding expression data set via the UCSC gene browser. We then describe how we implemented this in a way that can handle data sets much larger than those available for the present study. As:

1. quantitative measures of gene expression become more accurate,

2. as gene terminology become standardized across genomes,

3. as data on multiple tissues are generated, and

4. as we compare more highly rearranged genomes,

it will be useful to have a high throughput system to generate the data for statistical analysis.

##### Link breakpoints and expression via gene names

The protocol is as follows.

1. Scan the gene expression database for genes showing significant fold change in the human-macaque or human-chimpanzee comparison and extract the human gene name.

2. Locate the records for these differentially expressed genes in the genome browser, by matching names in the two databases. This step is not fully automated since a good proportion of the "names" in the whole blood tissue expression database are not gene names at all, but are ESTs or transcripts of part of the gene, which can be located in other UCSC browser files, or obsolete gene names, which have to be tracked down by web search. A full 50 of the 317 differentially expressed elements in the human-macaque study did not have hits at all in the UCSC browser, and had to be dropped from our analysis. Eight of the 80 differentially expressed genes in the cerebral cortex study were discarded for the same reason.

3. Extract the chromosome and coordinates of these genes in the human genome, and in the case of the cerebral cortex study, in the chimpanzee genome.

4. Compute the distance in nucleotides to the closest BPR in the BPR database.

5. Similarly, for all the human genes in the full genome browser that do not match those differentially expressed genes previously identified, compute the distance in nucleotides to the closest BPR.

Having extracted all these data on 267 differentially expressed genes from the whole blood tissue expression database, or the 72 differentially expressed genes from the cerebral cortex expression database, as well as the corresponding information on the rest of the human gene complement, we are now in a position to treat them statistically.

##### Implementation

We designed a relational database schema, implemented in PostgreSQL [17], to integrate the three different kinds of dataset: BPRs, differentially expressed genetic elements and all human genes. We loaded this with the data described in the preceding **Breakpoint data**, **Gene expression data **and **Genome database **sections. We also loaded UCSC browser counterparts of the mRNA and EST entries found in the differentially expressed genes database. In addition, we loaded the entire set of human genes from the UCSC Known Genes Table. For the cerebral cortex study, we loaded the entire set of chimpanzee genes from the chimpanzee genome assembly of March, 2006 (CHIMP2.1) and the human genome assembly of February, 2009 (NCBI37 or GRCh37) from the Ensembl Genome Browser [16] into the genome database.

We queried the relational database with a series of SQL statements implementing the different steps described in the preceding Section in order to link the information and compute the distance *d *between each differentially expressed gene and its closest BPR, as well as the distance between each gene in the remainder of the human gene complement, and its closest BPR.

## Results of analyses

### Whole blood tissue

**Figure 3:**
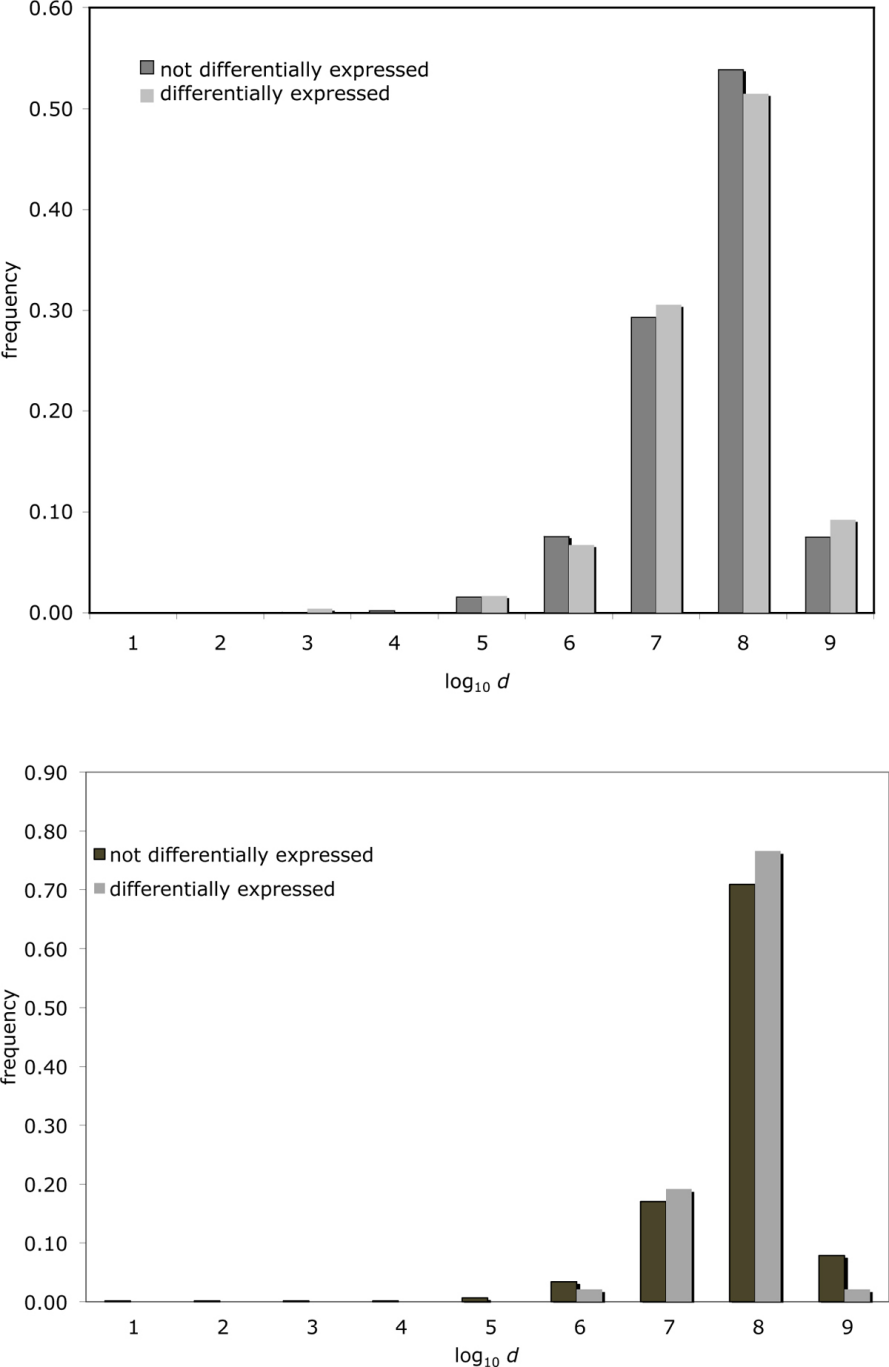
**Differential expression and distance to breakpoint.** Histogram of distances from genes to closest BPR for differentially expressed genes vs. not differentially expressed genes for all chromosomes. Top: whole blood tissue. Bottom: cerebral cortex tissue.

Figure 3(top) compares the distance to the nearest breakpoint of differentially expressed genes to that of the entire set of human genes located in all of the chromosomes that contains breakpoints. While the shape of the distribution is generally as expected from our model illustrated in Figure 1, it is clear that there is little difference between the distributions for the differentially expressed genes and the rest of the human gene complement. This is what we would expect if rearrangement generally has no impact on gene expression. However, this does not mean that rearrangement never has this effect.

**Figure 4:**
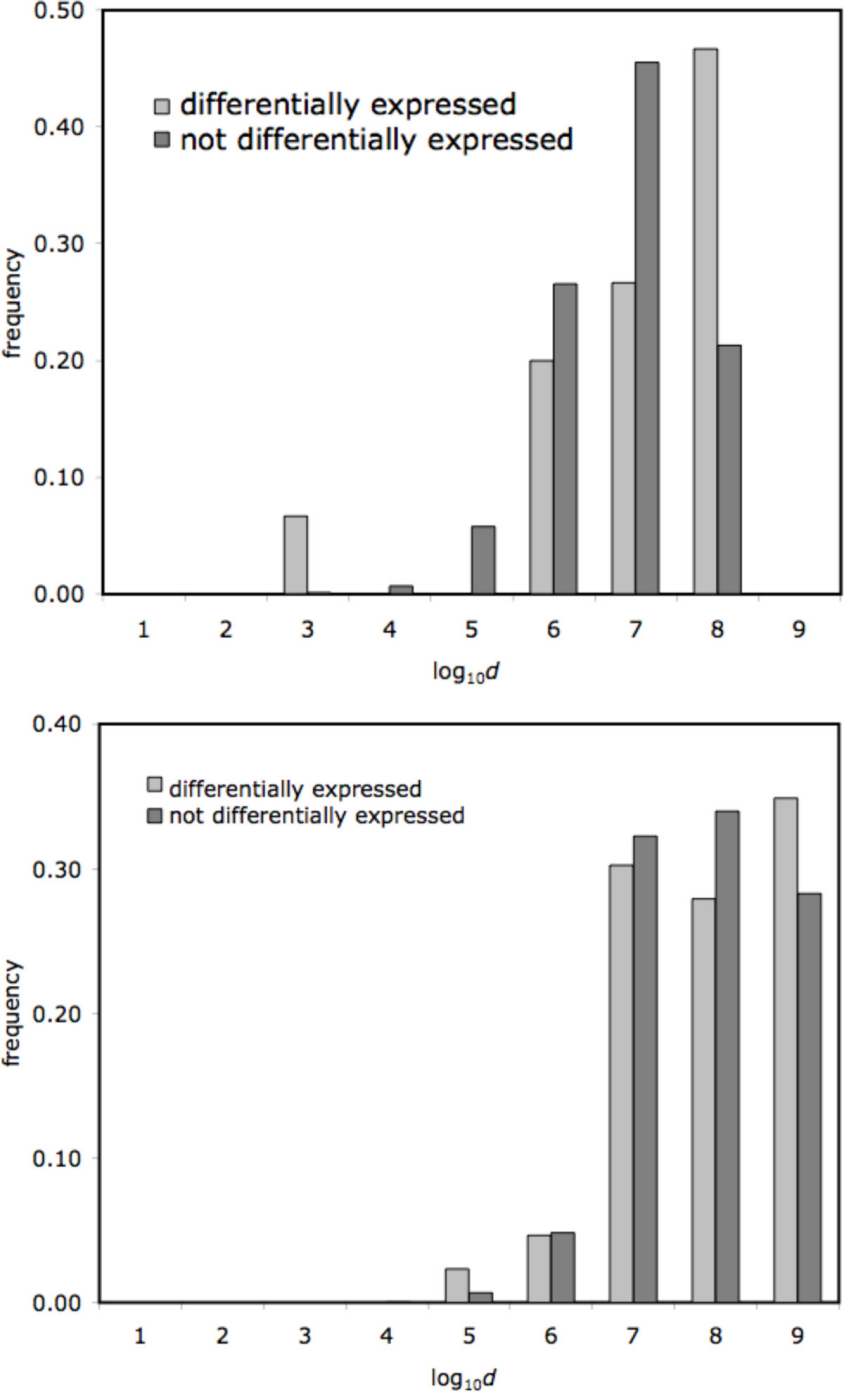
**Expression in blood and distance to breakpoint for two chromosomes.** Histogram of distances from genes to closest BPR for differentially expressed genes vs. not differentially expressed genes for chromosome 16 (top) and for chromosome 1 (bottom) for whole blood tissue.

This prompted us to inspect more closely the small number of differentially expressed genes close to BPRs for each chromosome that contains breakpoints: one in chromosome 16 where *d *= 216 and four in chromosomes 1,2 and X with *d <*10^5^. As a visualization tool, our distribution on a log scale depicts this neatly, as in Figure 4. for genes in chromosome 16 and chromosome 1.

#### NBPF

Though the few differentially expressed genes close to breakpoints that we found do not seem to be functionally related, it is of interest that one of those on human chromosome 1 is member 10 of the Neuroblastoma breakpoint family (NBPF). This family was so named because of a patient with a constitutional translocation t(1;17)(p36;q12-21) breakpoint near a gene family member, thought to suppress formation of this tumour, eventually developed a neuroblastoma [18]. This gene family is known to evolve rapidly in the primates, by full and partial duplication and divergence [19], has undergone a rapid recent expansion reflected in copy number variation in humans [20], and is thought to play a role in the physiological divergence of primate species. Thus it is of particular interest that one family member near an evolutionary breakpoint has changed expression level in the whole blood tissue study.

### Cerebral cortex study

As in the blood study, Figure 3 (bottom) compares the distance to the nearest breakpoint of differentially expressed genes to that of the entire set of chimpanzee genes located in one of the chromosomes that contains breakpoints, in the cerebral cortex data. Again the shape of the distribution is as expected from our model illustrated in Figure 1, so that there is little difference between the distributions for the differentially expressed genes and the rest of the human gene complement.

**Figure 5:**
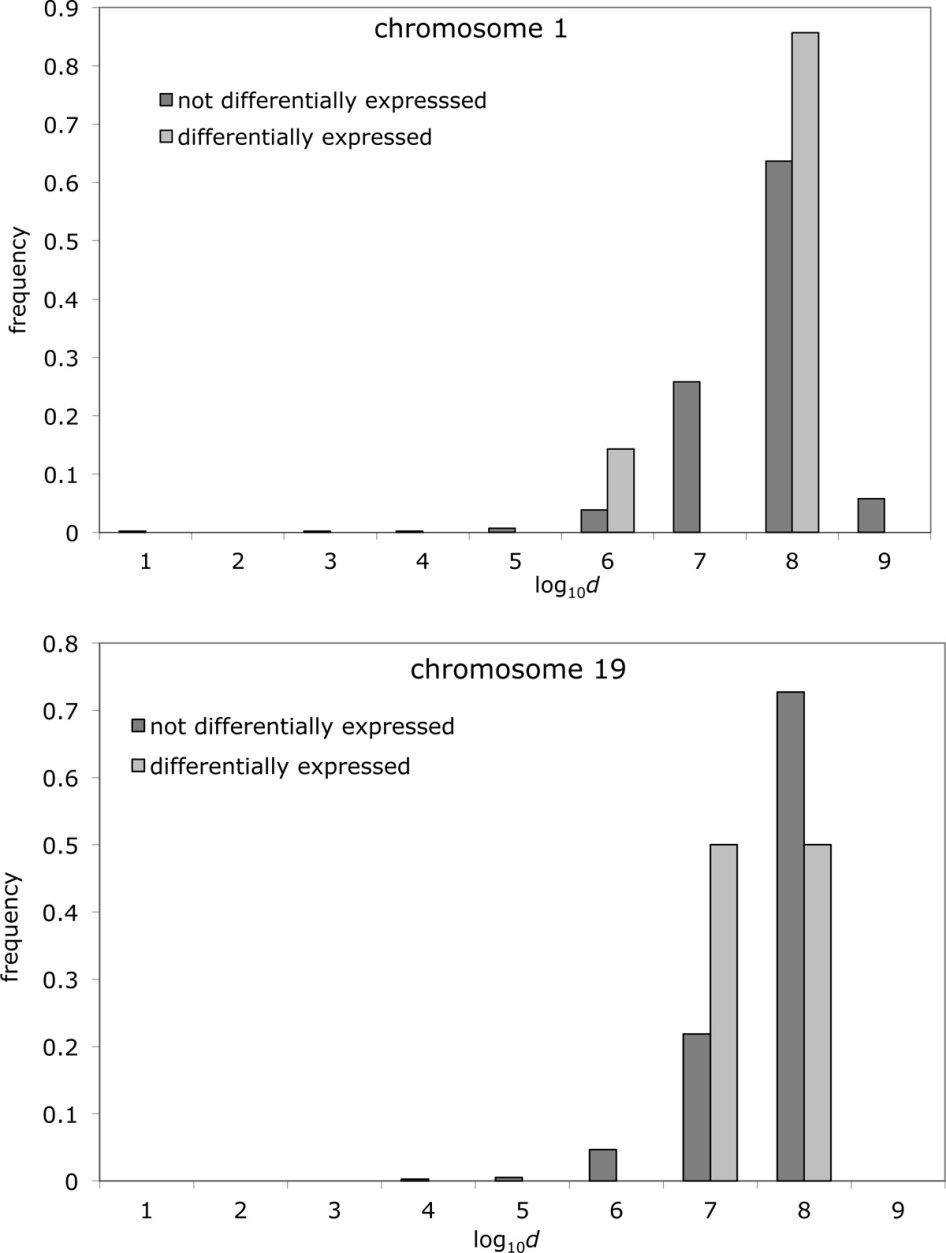
**Expression in brain and distance to breakpoint for two chromosomes.** Histogram of distances from genes to closest BPR for differentially expressed genes vs. not differentially expressed genes in cerebral cortex tissue for chromosome 1 (top) and for chromosome 19 (bottom).

Again, however, we examined a number of differentially expressed genes close to BPRs in chimpanzee for each chromosome that contains breakpoints: one in chromosome 1 where *d *= 9.5 Kbp and four in chromosomes 19, 15, and 17 where *d *between *d *= 2.2 and *d *= 3.2 Mbp. As a visualization tool, our distribution on a log scale depicts this neatly, as in Figure 5 for genes in chromosome 1 and chromosome 19 in the chimpanzee genome. It is interesting to note the two closest differentially expressed genes to a BPR in chromosome 19 shared the same BPR where *d *= 2.2 and *d *= 2.6 Mbp, respectively (see Figure 5, bottom).

#### MAPT

The *MAPT *gene was the closest differentially expressed gene to a breakpoint in chromosome 17 in the chimpanzee vs. human comparison, where *d *= 3.2 Mbp. This gene codes for Tau, a protein involved in the nucleation, elongation, and stabilization of microtubules [21]. It is associated with a group of human neurodegenerative diseases characterized by the presence of filamentous Tau deposits in nerve cells and glial cells [21,22], such as Alzheimer's disease (AD), progressive supranuclear palsy (PSP), and frontotemporal dementia and parkinsonism linked to chromosome 17(FTDP-17)). The chimpanzee brain has a relative resistance to developing Tau pathology [23]. Since humans and great apes have very similar Tau protein sequences, differences in intronic sequence might explain their differential susceptibility to developing filamentous Tau inclusions, in particular, the apparent resistance of the chimpanzee to developing a filamentous Tau pathology in the brain [24]. The proximity of the *MAPT *gene to an evolutionary breakpoint that we have pointed out here, in connection with its changed expression level in chimpanzee brain tissue, suggests that the wider chromosomal environment of the gene may also play a role in the resistance of the chimpanzee to developing Tau pathologies.

#### The retinoblastoma genes

The Retinoblastoma 1 gene *RB1 *on chromosome 13 regulates cell growth and proliferation in the brain and other organs, and the suppression of both copies of this gene is associated with an embryonic neoplasm of retinal origin called retinoblastoma. A study of the high degree of sequence conservation of RB1 in human and primates supports a hypothesis of purifying selection in *RB1 *throughout the history of primates [25].

We found that the Retinoblastoma-like 2 (*RBL2*) gene was the closest differentially expressed gene to a breakpoint in chromosome 16 in the chimpanzee vs. human comparison, where *d *= 7 Mbp. This gene, down-regulated in chimpanzee, regulates *RB1*.

In addition, in our survey, we found that the retinoblastoma-binding protein 5 (*RBBP-5*) was the closest differentially expressed (up-regulated) gene to a breakpoint in chromosome 1 in the same comparison, where *d *= 9.5 Kbp.

The facts that both *RBL2 *and *RBBP-5 *interact with the highly conserved *RB1*, and that both change expression consequent to rearrangement events, suggest a possible role of the rearrangement process in concert with purifying selection processes, in maintaining or adjusting the function of *RB1*.

## Conclusions

Genome rearrangement research has been concerned with the creation of breakpoints and their position in the chromosome. The question arises of what the biological consequences of breakpoint creation are, rather than just their structural aspects.

Since a chromosomal rearrangement may occasionally disrupt the spatial connection between a gene and its regulatory regions, we have asked whether proximity to the site of a breakpoint event changes the activity of a gene. We investigated this by comparing the distribution of distances to the nearest breakpoint of genes that change expression after rearrangement with the same distribution for those that do not change. This question has not been investigated previously on a genome-wide basis.

The data currently available on individual gene expression change across entire genomes for different species is limited. That we found little evidence for rejecting the null hypothesis is attributable to sparse data and to relatively crude measures of fold changes. With the advent of Next Generation Sequencing, quantitative RNA sequence data on many tissues from related species should soon become available. Our computational pipeline may be of utility at that time.

## Competing interests

The authors declare that they have no competing interests.

## Authors' contributions

AM and DS formulated the problem. AM designed and implemented this computational pipeline in Java, PostgreSQL 8.4.3 [17] relational database, SQL, R, and MS Excel. AM selected the two gene expression studies, extracted the data sets from the public databases and loaded them into our PostgreSQL database. AM conducted the interpretation of the results. AM and DS contributed equally to the writing of this manuscript.
